# Evaluation of the effect of autologous conditioned serum on the radiographic characteristics of hard tissue after horizontal bone augmentation in implant dentistry

**DOI:** 10.34172/japid.2022.015

**Published:** 2022-10-26

**Authors:** Hamidreza Mohammadi, Adileh Shirmohammadi, Amirreza Babaloo, Leila Roshangar, Zeinab Torab, Mehdi Mojtahedinia

**Affiliations:** ^1^Department of Periodontics, Faculty of Dentistry, Tabriz University of Medical Sciences, Tabriz, Iran; ^2^Department of Histology and Embryology, Faculty of Medicine, Tabriz University of Medical Sciences, Tabriz, Iran; ^3^Department of Pediatric Dentistry, Faculty of Dentistry, Tabriz University of Medical Sciences, Tabriz, Iran

**Keywords:** Alveolar Ridge Augmentation, dental Implant, novel Interleukin Receptor

## Abstract

**Background.** Bone deficiency in different areas is problematic in implant placement. Changes in histological, histomorphometric, and radiographic properties of hard tissues in the implant placement area affect many parameters of implant success. Autologous conditioned serum (ACS) is a blood product with high levels of IL1- receptor antagonists. Augmentation surgeries are required in many cases because implant placement in the edentulous areas requires a sufficient amount of bone. Therefore, this study radiographically evaluated the effect of autologous conditioned serum after horizontal bone augmentation.

**Methods.** In this prospective RCT, 21 patients eligible patients were evaluated. The patient underwent horizontal ridge augmentation surgery in the area. The ACS-impregnated graft was in direct con­tact with the bone. The control side underwent the same surgical protocol without using ACS. Four months after surgery, a CBCT radiograph was taken, and radiographic changes in the two areas were calculated using the differences in the amount of bone formed in the horizontal dimension as well as the Hounsfield unit (HU). The data were reported using descriptive statistical methods, including means (standard deviations) and frequencies (percentages). According to the results of the Kolmog­orov-Smirnov test, the data had a normal distribution (P>0.05); therefore, paired t-test was used to compare the means of the parameters between the two groups.

**Results.** IRadiographic examinations showed that the horizontal dimension of bone before surgery was similar between the two groups. However, after surgery in the ACS group (33.13±6.1), it was significantly higher than in the control group (62.1±86.4) (P>0.05). Also, the rate of horizontal dimension increase (the difference before and after surgery) in the ACS group was significantly higher than in the control group. Bone density before surgery was similar between the two groups. However, after surgery, there was a significant increase in the ACS group (75.56±330.42 HUs) compared to the control group (38.35±292.38 HUs) (P>0.05). Also, the rate of density increase (the difference before and after surgery) in the ACS group was significantly higher than in the control group.

**Conclusion.** Radiographic evaluations of hard tissues showed a significant increase in the horizontal dimension of bone and density of newly formed bone using ACS compared to the control group.

## Introduction

 Many traumatic events, including tooth loss, sinus pneumatization, periodontal disease, etc., lead to bone loss in different areas of the alveolar ridge.^1^Bone deficiency in different areas is problematic in implant placement, necessitating horizontal bone augmentation and socket preservation methods to improve hard tissue conditions in the area.^2^Changes in histological, histomorphometric, and radiographic properties of hard tissues in the implant placement area affect many parameters of implant success.^3^Improving characteristics such as alkaline phosphatase enzyme activity, the rate at which new viable bone is formed, and its pattern of mineralization and trabeculae are essential for achieving a high success rate for implants placed in the area.^3^

 As a potent inflammatory mediator, IL-1 is involved in many of the body’s inflammatory processes and is responsible for many restorative outcomes of therapeutic interventions in the implant area.^4^This mediator is released in physiological amounts from different cells, such as macrophages in the area, and affects the progression of inflammation and ultimate tissue repair.^5^However, in high amounts, it exacerbates inflammation and destructive processes, including bone loss.^4^It seems necessary to control and modulate the host’s inflammatory responses in situ to affect the secretion of various cytokines and activate or inhibit their activity, depending on the time and place of intervention, reduce the destructive processes, and improve tissue properties. Autologous conditioned serum (ACS) is a blood product with high levels of IL-1 receptor antagonists. The IL-1 receptor antagonist is also found naturally in the body. ACS was first used as a new therapeutic agent in the mid-1990s to treat osteoarthritis in injectable form with high levels of IL-1 receptor antagonists. This product is used topically to treat and improve bone resorption and inflammation of the area where IL-1 is the primary etiologic agent. ACS is used to treat degenerative joint diseases, especially knee osteoarthritis.^5^ Today, prefabricated forms of this product are also available, and it has become very popular for treating degenerative joint events.^6^This product seems useful and necessary for periodontal regenerative processes and implantation site enhancement. In a study, injecting an IL-1β antagonist to treat arthritis in rats and humans inhibited the IL-1Ra receptor, preventing the destruction of the extracellular matrix of chondrocytes.^7^One study examined the effect of commercially available ACS on temporomandibular joint inflammatory disorders and reported promising results in treating TMJ arthritis.^8^Another study reported that injecting commercially available ACS to treat arthritis decreased joint pain and improved its function. They attributed this improvement in joint function to decreased inflammation and reduced joint damage.^9^

 Augmentation surgeries are required in many cases because implant placement in the edentulous areas requires a sufficient amount of bone. The bone in many edentulous ridges is dimensionally deficient and problematic or will be problematic in the future.

 This material appears to be useful in procedures to regenerate bone defects and enhance histologic characteristics. It is suggested that more studies be carried out in this field to achieve the mentioned goals.

 Cone-beam computed tomography (CBCT) provides a three-dimensional image of dental and maxillofacial areas. CBCT is a useful tool for locating anatomical structures, supporting diagnostic implant planning, and guiding dental surgeries. Reliable linear measurements of dentomaxillofacial structures can be made using a CBCT image. Therefore, CBCT can potentially evaluate healing after alveolar ridge preservation procedures.^10,11^

 Several studies have evaluated the accuracy and reproducibility of linear measurements made on cone-beam computed tomographic images. Loubele et al^12^ compared the accuracy of linear measurements of alveolar bone taken on Accuitomo CBCT images, multislice CT images, and direct measurements on an ex vivo model and found no significant differences between any of the measurement methods, later confirmed by Lund et al,^13^ who also reported a high level of agreement between measurements made by multiple observers.

 Tomasi et al^14^reported a high correlation between linear measurements made with a caliper and measurements made on a CBCT image. However, Torres et al^15^ found that typical CBCT images underestimate the actual linear distance by 16‒17% in the horizontal plane and 7‒8% in the vertical plane. Hashem et al^16^ found no differences between two different methods of capturing a CBCT image compared to direct measurements made on porcine hemi-mandibles. Shokri et al^17^ found a high level of agreement between actual measurements with a digital caliper and measurements made on CBCT. They reported that the most accurate horizontal measurements were made with slice thicknesses of 4‒5 mm. They concluded that measurements made on slices <4 mm might underestimate the actual distance.

 CBCT obtains images using a two-dimensional detector to scan the entire head, rather than stacking multiple slices as in conventional computed tomography, allowing for a more efficient, less expensive, and lower energy output image.^18,19^ CBCT does not use high radiation doses. The average radiation dose of 0.585 mSv is well below that of a conventional medical-grade CT scan but above that of conventional dental radiographs. Cortical integrity and thickness, enlarged bone marrow spaces, post-extraction irregularities, and trabecular bone density are identified clearly in the cross-sectional images produced by the CBCT.^20^

 This study radiographically evaluated the effect of autologous conditioned serum after horizontal bone augmentation.

## Methods

###  Study design and patient selection

 The present prospective RCT was conducted according to the 1975 Declaration of Helsinki and its amendments from 2000. This study was approved by the Ethics Committee of Tabriz University of Medical Sciences (Approval Number: IR.TBZMED.REC.1400.291). The study enrolled 21 patients who visited the Department of Periodontics, Faculty of Dentistry, Tabriz University of Medical Sciences, from May 2019 to January 2020. The patients required cone-beam computed tomography (CBCT) examinations twice, and 10 mL of their blood sample was collected. All the enrolled patients signed an informed consent form before participating in the trial. Eligible patients included those scheduled for implantation.

**Figure 1:**
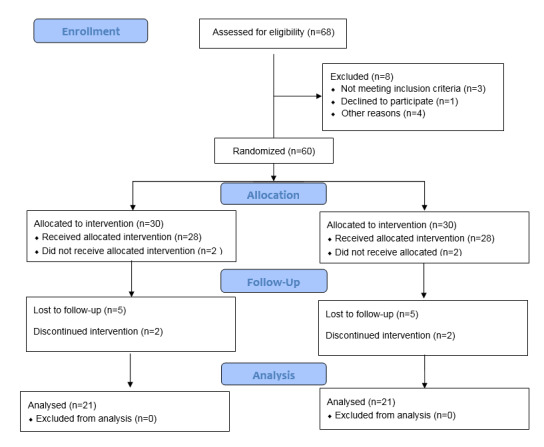
Consolidated Standards of Reporting Trials Diagram.

 To improve the reporting of RCT, we followed the Consolidated Standards of Reporting Trials Diagram (Figure 1).

###  Exclusion and inclusion criteria

 The inclusion criteria: The difficulty of surgery was the same as the other side based on the dimensions required for augmentation and ridge conditions; patients required implant placement in the area and were willing to undergo surgery.

 The exclusion criteria: Use of drugs that might interfere with treatment, systemic diseases, smoking, inflammation or infection or pain during surgery, a recent history of nonsteroidal inflammatory drug use, patients with gastrointestinal problems, NSAID allergies, and pregnancy.

###  Study protocol

###  Autologous conditioned serum preparation

 One day before surgery, 10 mL of blood was taken from the patient’s vein and immediately injected into a special ACS syringe. The syringe was incubated at 37°C for 8 hours and then centrifuged at 3000 rpm for 10 minutes. After the centrifugation stage, the syringe was removed from the device, which had two upper and lower parts. The upper part was carefully removed, stored as ACS in the refrigerator, and used on the day of surgery.

###  Horizontal bone augmentation procedure

 The patient underwent horizontal ridge augmentation surgery in the area after injection of local anesthesia and ensuring anesthesia. The incision was made in the area using a #15 blade, and the tissue was elevated using a periosteum retractor. The area was prepared for placing a freeze-dried bone material (250-100µm/2cc/Regen Inc./Iran) and ACS-impregnated membrane (Acellular Dermis Membrane/1.1-1.4mm thickness/2*4mm/ Regen Inc./Iran). The buccal cortex was decorticated by a milling process at 2-mm intervals, and the ACS-impregnated graft was in direct contact with the bone.

 A collagen membrane with a thickness of 1.1‒1.4 was impregnated with 1 mL of prepared ACS and placed in the area. The elevated soft tissue was returned and sutured using 3-0 silk. The control side underwent the same surgical protocol without using ACS.

 The necessary medications were prescribed, including chlorhexidine mouthwash, acetaminophen, and antibiotics, and the patient was provided with the necessary recommendations after surgery.

###  Radiographic evaluation

 Four months after surgery, the area for implant placement was evaluated. A CBCT radiograph was taken, and radiographic changes in the two areas were calculated using the differences in the amounts of bone formed in the horizontal dimension as well as the Hounsfield unit (HU). A comparison of postoperative samples (membrane area and graft material in the ACS-impregnated group and membrane group alone) was performed for these two variables.

###  Data analysis

 The data were reported using descriptive statistical methods, including means (standard deviations) and frequencies (percentages). Paired t-test or a non-parametric equivalent test was used to compare the amount of bone formed in the study group. According to the Kolmogorov-Smirnov test, the data had a normal distribution (P>0.05); therefore, paired t-test was used to compare the means of the parameters between the two groups. To compare bone width and bone density between the control and test groups, the values ​​before surgery should not be significantly different between the two groups; therefore, the differences in the means of these parameters after surgery between the two groups can be attributed to the intervention performed. This comparison was performed using paired t-test. This condition was met, and the mean of preoperative measurements between the two groups was not statistically significant (P=0.706).

 All the tests were carried out using SPSS 21. In this study, P<0.05 was considered significant. It should be noted that the Hounsfield unit was used to investigate bone density with the help of Mimics 10.01 software.

## Results

 In this double-blind clinical trial with a split-mouth design, 21 candidates for implants were examined. In each patient, an ACS-impregnated membrane and a bone graft material were used on one side, and bone membrane and powder alone on the other side.

 The results are presented in Table 1. Radiographic examinations showed that the horizontal dimension of bone before surgery was similar between the two groups. However, after surgery, in the ACS group (6.33±1.13), it was significantly higher than in the control group (4.86±1.62) (P=0.028). Also, the rate of horizontal dimension increase (the difference before and after surgery) in the ACS group was significantly higher than in the control group. Bone density before surgery was similar between the two groups. However, after surgery, there was a significant increase in the ACS group (330.75±42.56 HUs) compared to the control group (292.38±38.35 HUs) (P=0.031). Also, the rate of density increase (the difference before and after surgery) in the ACS group was significantly higher than in the control group.

**Table 1:** Comparison of horizontal bone dimensions and bone density after horizontal ridge augmentation surgery in the control and ACS groups

**Variables**	**Study Group**	**No.**	**Before surgery**		**After surgery**		*P-value	**Mean difference before and after surgery**	
			**Mean**	**SD**	**Mean**	**SD**		**Mean**	**SD**
**Horizontal bone dimension**	**ACS**	21	2.85	1.09	6.33	1.13	<0.001	3.48	1.11
	**Control**	21	2.73	1.03	4.86	1.62	<0.001	2.13	1.32
**P-value**			0.822		0.028			0.048	
**Bone density**	**ACS**	21	274.68	20.64	330.75	42.56	<0.001	56.07	31.62
	**Control**	21	271.39	21.92	292.38	38.35	<0.001	20.99	30.13
**P-value**			0.863		0.031			<0.001	

P-value: Paired-samples t-test (a comparison of the two groups) ^*^P-value: Paired-samples t-test (comparison of a group before and after surgery)

## Discussion

 A narrow alveolar ridge is a challenging condition for surgeons during implant placement. Different surgical methods, different materials, and choosing the best ridge augmentation method are still a challenge in implantology. The autologous conditioned serum is widely used in orthopedics and sports medicine to relieve pain through the natural healing of musculoskeletal disorders such as tendonitis, arthritis, ligament sprains, and tears. However, it has not been used in maxillofacial bone reconstruction surgeries. The present split-mouth study investigated the effect of ACS on histological and radiographic characteristics of hard tissues after horizontal ridge augmentation surgery and compared them with a control group.

 The bone graft should be able to induce a sufficient volume of ossification. A review of various studies shows that most of the existing bone graft compounds can produce bone in the 14‒44% range.

 Studies have shown that platelet-rich blood derivatives (PRP, PRF) or growth factors (CGF) can improve bone density. In this regard, Kolerman et al^21^ showed the acceleration of cartilage differentiation of DPSC using PRP, and Lee et al^22^ showed that the appropriate concentration of PRP increased the proliferation and differentiation of human dental stem cell colonization. In addition, Zhang et al^23^ and Kolerman et al^21^ showed the positive effects of PRP and PRF on new bone formation in sinus lift procedures.

 Histometric and radiographic results of Kim et al^24^ showed that PRP, PRF, and CGF increased the success rate of bone grafts and bone healing in animals. Acceleration of osseointegration and an increase in osteogenic proliferation and differentiation by concentrated growth factors (CGF) have also been confirmed in studies by Pirpir et al^25^ and Khurana et al^26^ Due to the structural and functional similarities between ACS and PRP, the results of the mentioned studies were confirmed in the present study. In addition, due to the cell-free nature of ACS, it is expected to cause fewer allergic reactions compared to PRP and other similar blood products.^1^

 In this regard, Shirokova et al^27^ showed the superior clinical and biochemical efficacy of ACS over PRP in treating osteoarthritis. Darabos et al^28^ identified the epidermal growth factor in ACS.

 A review by Shakouri et al^29^demonstrated the ability of ACS to target the inflammatory cascade to reduce degradation and improve endogenous repair mechanisms in degenerative joint disease.

 There are several methods for evaluating bone regeneration, such as bone strength, percentage of regenerated bone, biomechanical evaluation, and CT scans. In the present study, radiographic assessments of hard tissues after horizontal ridge reconstruction surgery were performed using CBCT, which showed significant effects of ACS on ossification, such as the increase in horizontal bone dimension (mm) and bone density (Hounsfield unit) in the study group. The control group values were 2.13 and 20.99, respectively, while in the autologous concentrated serum group, these values ​​increased to 3.48 and 56.07, respectively.

 Using CBCT radiographs, Thanasrisuebwong et al^30^showed that in vertical and horizontal bone regeneration, L-PRF and i-PRF provided biologically active and scaffold molecules for osteogenesis. Therefore, this treatment protocol might be a viable option because it is a large bone defect that should be reconstructed before implant placement.

 Since new bone formation is an important indicator in horizontal ridge augmentation surgery, the increase in bone formation in the ACS group was due to the presence of growth factors and cytokines, increased metabolism, and intensification of angiogenesis and collagen production, leading to more new bone formation.

 Velloso et al^31^showed that when PRP is applied topically to bone fracture sites, more regular remodeling and homogeneous bone density are obtained, similar to normal bone tissue.

 In a study by Valladão et al^32^on horizontal and vertical bone augmentation in implants using the GBR method, PRF showed an increase in bone thickness and height after 7.5 months.

 However, in a study by Lacheta and Braun,^33^ the use of orthokine in patients with knee osteoarthritis, the knee did not delay the surgical treatment time.

 Simion et al^34^ examined the increase in vertical ridge height in adult wolves and observed increased osteogenesis in samples containing platelet-derived growth factors.

 It can be concluded from the results of the present study that horizontal ridge augmentation by ACS is reliable for implant placement. However, further studies are necessary.

## Conclusions

 Radiographic evaluations of hard tissues showed a significant increase in the horizontal dimension of bone and density of newly formed bone using ACS compared to the control group.

## Acknowledgments

 We would like to acknowledge the Department of Periodontics, Faculty of Dentistry, for their assistance.

## Competing Interests

 The authors declare that they have no competing interests concerning the authorship and/or publication of this paper.

## Authors’ Contributions

 AS prepared the proposal, set and entered the results of the studies and interpreted them, prepared the final re­port and results, and wrote the manuscript. HM and ZT super­vised the design and execution of the study and prepared the final report. LR contributed to the preparation of the proposal, conducted the research, and collected the data. All authors approved the final manuscript.

## Funding

 This work was supported by the Vice-Chancellor for Research, Faculty of Dentistry, Tabriz University of Medical Sciences, Tabriz, Iran.

## Availability of data

 The raw data from the reported study are available upon request from the corresponding author.

## Ethics Approval

 This research was approved by the Research Ethics Committee of the Faculty of Dentistry under the code IR.TBZMED.REC.1400.291.
