# Correction to: Evaluation of the effect of autologous conditioned serum on the radiographic characteristics of hard tissue after horizontal bone augmentation in implant dentistry

**DOI:** 10.34172/japid.2023.013

**Published:** 2023-06-14

**Authors:** Hamidreza Mohammadi, Adileh Shirmohammadi, Amirreza Babaloo, Leila Roshangar, Zeinab Torab, Mehdi Mojtahedinia

**Affiliations:** ^1^Department of Periodontics, Faculty of Dentistry, Tabriz University of Medical Sciences, Tabriz, Iran; ^2^Department of Histology and Embryology, Faculty of Medicine, Tabriz University of Medical Sciences, Tabriz, Iran; ^3^Department of Pediatric Dentistry, Faculty of Dentistry, Tabriz University of Medical Sciences, Tabriz, Iran

 In the article entitled “Evaluation of the effect of autologous conditioned serum on the radiographic characteristics of hard tissue after horizontal bone augmentation in implant dentistry” which appeared in *J Adv Periodontol Implant Dent*. 2022;14(2): 62-68,^1^ typesetting errors have occurred. The mean and standard deviation numbers have been mixed in the results section of the abstract. Also, in the competing interests section of the article, the sentence stating the competing interests of Dr. Adileh Shirmohammadi as the editor-in-chief of JAPID at the time of publication was mistakenly omitted in typesetting. The journal regrets these errors. The original article has been updated to reflect these corrections.
